# Expression of follicle stimulating hormone receptors (FSHR) in thyroid tumours – a marker of malignancy?

**DOI:** 10.1186/s13044-015-0014-6

**Published:** 2015-02-05

**Authors:** Marek Pawlikowski, Julita Fuss-Chmielewska, Maria Jaranowska, Hanna Pisarek, Robert Kubiak, Katarzyna Winczyk

**Affiliations:** Department of Immunoendocrinology, Chair of Endocrinology, Medical University of Lodz, Lodz, Poland; Department of Neuroendocrinology, Chair of Laboratory Medicine, Medical University of Lodz, Lodz, Poland; Department of Pathology of Tumors, Chair of Oncology, Medical University of Lodz, Lodz, Poland

## Abstract

**Background:**

In normal conditions FSHR are expressed in granulosa cells of the ovary and Sertoli cells of the testis. They can be expressed also in gonadal tumours. However, recently the expression of FSHR was found in tumoral cells and intra-tumoral blood vessels of many other tumours, including thyroid tumours. Aim of this study was to see whether the expression of FSHR can be useful in the differentiation of benign and malignant thyroid lesions.

**Methods:**

44 samples of surgically excised thyroids were immunostained with anti- FSHR antibody raised against 1–190 amino acid sequence from the human FSHR.

**Results:**

Non-neoplastic thyroid follicles (i.e. the follicles situated outside the tumour) do not show the immunostaining for FSHR. The same concerns the majority of follicular adenomas. In contrast, 87.5% of follicular cancers, the same percentage of papillary cancers and all the examined undifferentiated cancers showed the FSHR immunopositivity of tumoral cells. A tendency towards the higher frequency of FSHR – positive blood vessels also concerns malignant thyroid tumours.

**Conclusions:**

The ectopic FSHR immunostaining seems to be useful to differentiate malignant from benign lesions, especially follicular cancers from follicular adenomas. However, the further studies on larger material are needed.

## Background

The follitropin receptors (FSHR) are normally expressed in granulosa cells of the ovary and in Sertoli cells of the testis. It is well known that FSHR can be also expressed in gonadal tumours [1]. Some years ago Radu et al. [2] reported on the ectopic expression of FSHR in the endothelium of intratumoral and peritumoral blood vessels in several malignant extra-gonadal tumours [2]. We confirmed this finding in endocrine tumours, including pituitary adenomas, adrenal benign and malignant tumours and neuroendocrine tumours of lungs and of the alimentary tract (carcinoids). Moreover, we also observed the positive immunostaining with anti-FSHR antibody in tumoral cells of the investigated tumours [3,4]. The latter observations corroborate with the earlier findings of Ben-Josef et al. [5] who reported on the FSHR expression in androgen-independent prostate cancer cells PC3 and DU145 and that of Sardella et al. [6] who found the expression of FSHR in the tumoral cells of neuroendocrine pancreatic tumours. The recent studies from our [7,8] and other [9] laboratory showed that the ectopic FSHR immunostaining is also present in thyroid neoplasms.

The aim of the study was to re-evaluate the possible usefulness of FSHR immunostaining to differentiate between benign and malignant thyroid lesions, especially between the follicular adenomas and follicular cancers.

## Methods

Forty four thyroid samples, obtained during surgery, were examined. The samples included 4 undifferentiated cancers, 16 papillary cancers, 16 follicular cancers and 8 follicular adenomas. FSHR immunostaining was performed on paraffin sections using the rabbit anti-human FSHR polyclonal antibody raised against 1–190 amino acid sequence from the human FSH-R (sc-13935, Santa Cruz Biotechnology Inc.). The primary antibody was applied in dilution of 1:100. The visualization of immunostaining was done using the Dako REAL EnVision Detection System (Dako-Cytomation, Denmark). The immunostaining intensity for FSHR in the tumoral cells was scored using a semiquantitative scale: negative staining (score 0), weak staining (score: 1), moderate staining (score: 2) and strong staining (score: 3). Only strong (score 3) or moderate (score 2) immunostaining was considered as meaningful. The slides stained with the omission of the primary antibody served as negative controls. The study was approved by the local ethical committee of the Medical University of Lodz, decision No RRN/151//14/KE.

## Results

**Figure 1:**
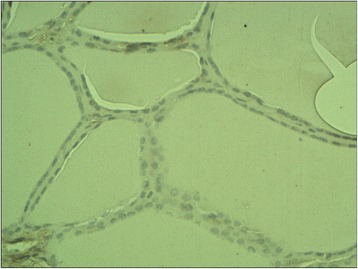
**Non-neoplastic thyroid epithelium.** Negative staining for FSHR. Final magnification 200×.

**Figure 2:**
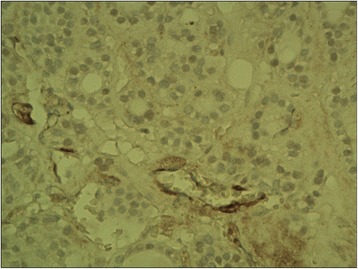
**Follicular adenoma.** Immunostaining with FSHR antibody negative in majority of cells, positive only in few cells. Final magnification 200×.

**Figure 3:**
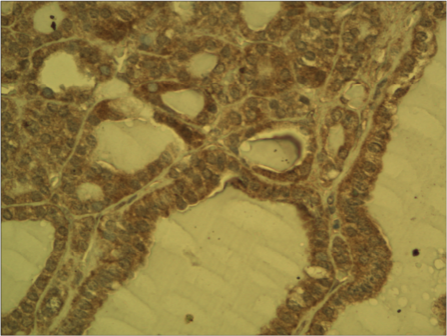
**Follicular cancer.** Immunostaining with FSHR antibody. Final magnification 400×.

**Figure 4:**
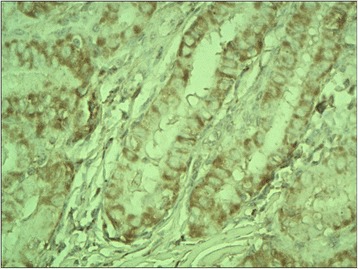
**Papillary cancer.** Immunostaining with FSHR antibody. Final magnification 200×.

**Figure 5:**
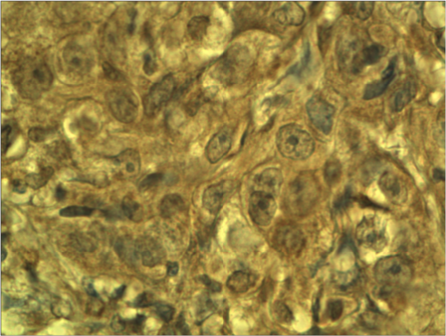
**Undifferentiated cancer.** Immunostaining with FSHR antibody. Final magnification 400×.

**Figure 6:**
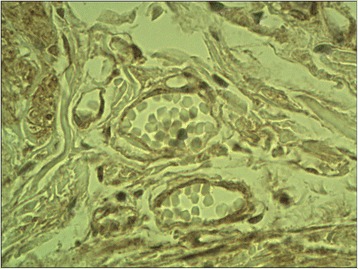
**Blood vessels immunopositive FSHR.** Follicular cancer. Final magnification 400×.

The ectopic FSHR immunostaining was absent in non-neoplastic thyroid follicles (Figure 1) (i.e. the follicles situated outside the tumour) and in the majority (all but two, Figure 2) of follicular adenomas. In contrast, the FSHR expression can be detected in tumoral cells of majority (88.8%, 32/36) of thyroid cancers. The positive FSHR immunostaining of tumoral cells was shown in 14 from 16 examined follicular cancers (87.5%, Figure 3). The same could be observed in 14 from 16 examined papillary cancers (87.5%, Figure 4) and in the all (4/4) undifferentiated cancers (Figure 5). The positive FSHR immunostaining is also present in the walls (mainly in endothelium) of some intra- or peri-tumoral blood vessels (Figure 6). The immunopositive blood vessels were detected in 3/8 (37.5%) follicular adenomas, 5/16 (31%) follicular cancers, 9/16 (56%) papillary cancers and in 3/4 (75%) undifferentiated cancers.

## Discussion

The observations presented above confirm that, like in the other human neoplasms, thyroid cancers in the great majority express FSHR in contrast to the non-neoplastic thyroid epithelium. The expression of FSHR may sometimes occur also in the benign thyroid lesions, like thyroid follicular adenomas but that is scarce. In contrast, Liu et al. [9] reported that FSHR expression is greater in thyroid adenomas than in papillary and poorly differentiated cancers. However, their material did not include the follicular cancers. The difference in FSHR expression between follicular cancers and follicular adenomas, as it was shown in the present study, seems to be of importance for a diagnosis. The differentiation among them by a pathologist on the basis of the morphological features is sometimes very difficult. Thus, the immunopositivity of FSHR could serve as an additional marker of malignancy. However, the further studies on larger material are needed in this respect.

There are many data concerning other tumours indicating the relationship of ectopic FSHR expression and malignancy grade. FSHR expression is observed in androgen-independent prostate cancer cells which are considered as less differentiated than androgen – dependent ones [5]. In liposarcomas, FSHR expression was observed in more cases of poorly differentiated than in well differentiated tumours. In contrast, benign lipomas and the normal fat is FSHR-negative [10]. In neuroendocrine tumours, the FSHR-positive blood vessels are more often present in the samples with higher proliferation index [4]. In pituitary adenomas, the expression of FSHR in tumoral cells is prevalent in invasive and proliferating, it means more aggressive tumours [11]. It remains unknown, whether the FSHR protein detectable in thyroid tumours is biologically active. If so, it may be also relevant for the outcome of tumours because, like in the ovary and ovarian cancers, FSHR may mediate the stimulatory effect of FSH on cell proliferation and angiogenesis [12,13] and the inhibitory effect on apoptosis [14].

## Conclusions

The ectopic FSHR immunostaining seems to be useful to differentiate malignant from benign thyroid lesions, especially follicular cancers from follicular adenomas. However, the further studies on larger material are needed.
